# Supplementary material for: The interactome of intact mitochondria by cross-linking mass spectrometry provides evidence for coexisting respiratory supercomplexes
Source: Mol Cell Proteomics. 2017 Dec 8;17(2):216–32. doi: 10.1074/mcp.RA117.000470 (PMC5795388; doi:10.1074/mcp.RA117.000470)
Supplement: Supplemental Data [file supp_RA117.000470_133922_0_supp_23978_fzffwf.zip › spectra_annotation/mito_spectra_annotation/171-1-4-1-12-1.pdf]

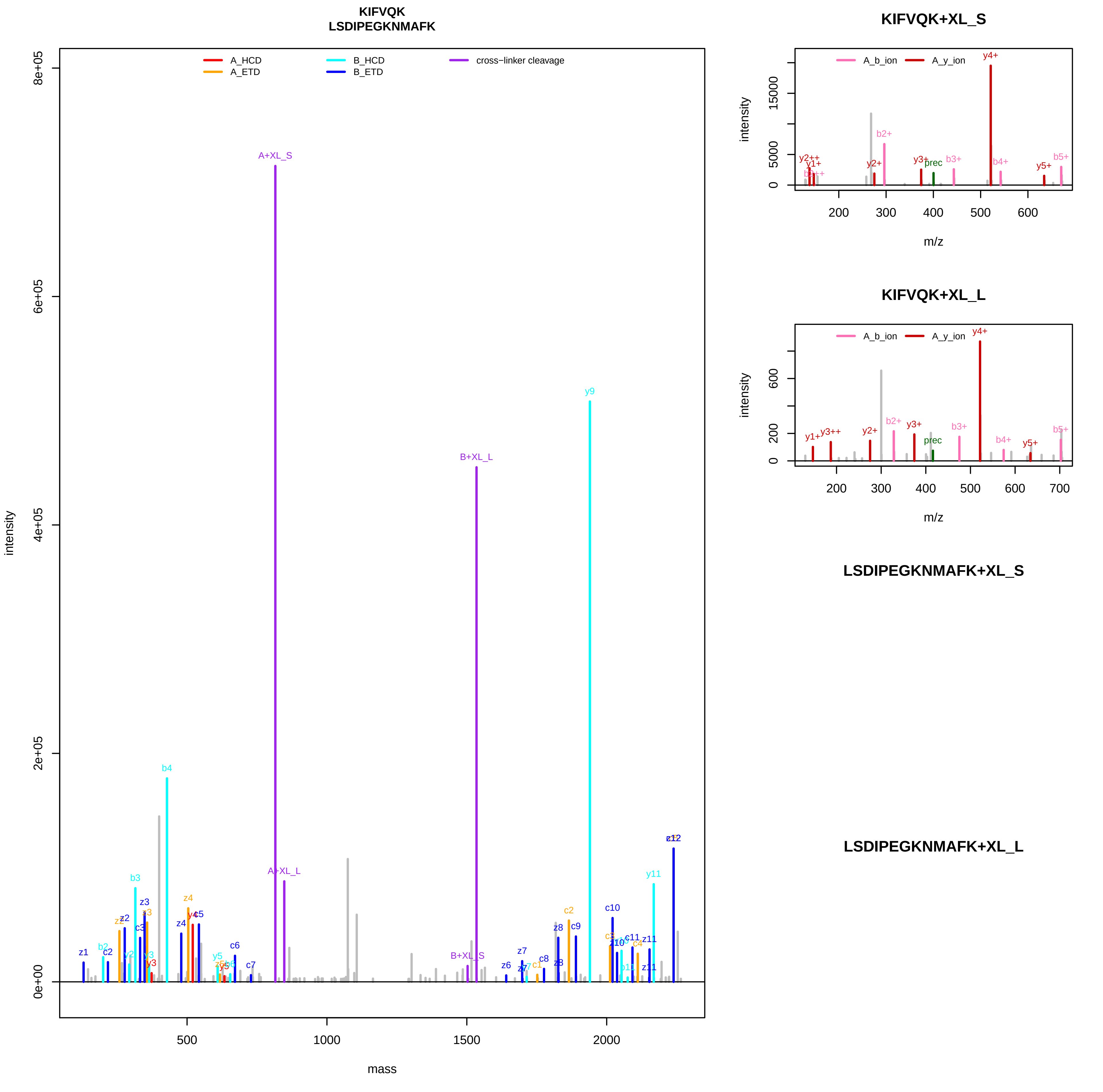

KIFVQK
LSDIPEGKNMAFK
A_HCD
A_ETD
B_HCD
B_ETD
cross−linker cleavage
A+XL_S
B+XL_L
A+XL_L
B+XL_S
y9
b4
b3
y11
c12
c10
c9
c2
z8
c11
z11
z1
c2
z2
c3
z3
z4
y4
c5
c6
c7
y5
z6
z7
c8
c1
c4
intensity
mass
0e+00
2e+05
4e+05
6e+05
8e+05
500
1000
1500
2000
KIFVQK+XL_S
A_b_ion
A_y_ion
y4+
b2+
y3+
prec
b3+
b4+
y5+
b5+
y2++
y1+
y2+
intensity
m/z
0
5000
15000
200
300
400
500
600
KIFVQK+XL_L
A_b_ion
A_y_ion
y4+
y1+
y3++
y2+
b2+
y3+
prec
b3+
b4+
y5+
b5+
intensity
m/z
0
200
600
200
300
400
500
600
700
LSDIPEGKNMAFK+XL_S
LSDIPEGKNMAFK+XL_L
